# Activation and gut-homing of peripheral T cells in HIV immunologic non-responders despite long term viral suppression

**DOI:** 10.1371/journal.pone.0254149

**Published:** 2021-07-28

**Authors:** Rodney K. Rousseau, Leah Szadkowski, Colin M. Kovacs, Michael F. Saikali, Rabea Nadeem, Fat Malazogu, Sanja Huibner, Carolyn L. Cummins, Rupert Kaul, Sharon L. Walmsley

**Affiliations:** 1 Department of Immunology, University of Toronto, Toronto, Canada; 2 Biostatistics Research Unit, University Health Network, Toronto, Canada; 3 Maple Leaf Medical Clinic, Toronto, Canada; 4 Department of Medicine, University of Toronto, Toronto, Canada; 5 Leslie Dan Faculty of Pharmacy, University of Toronto, Toronto, Canada; 6 Toronto General Hospital, University Health Network, Toronto, Canada; 7 Toronto General Hospital Research Institute, Toronto, Canada; University of Pittsburgh, UNITED STATES

## Abstract

**Objective:**

Serious non-AIDS disease events (SNAE) are experienced disproportionately by immunologic non-responders (INRs), HIV-infected individuals who do not restore CD4 T cells in blood despite effective viral suppression. We aimed to characterize the inflammatory biomarker profile of the INR phenotype.

**Methods:**

Blinded cross-sectional cohort study comparing markers of immune activation and gut homing between INR and non-INR individuals. HIV-positive participants had HIV RNA suppression on antiretroviral therapy and were categorized as either INR (N = 36) or Clinical Responders (“CR”; CD4>350/mm^3^; N = 47). 18 HIV-negative comparator individuals were included. Cellular markers were assessed by flow cytometry, with soluble markers assessed by ELISA and LC/MS-MS. Multivariable linear regression models estimated the association between INR phenotype and markers, adjusting for age, sex, duration of ART, and recent infection/vaccination.

**Results:**

INR participants demonstrated a reduced CD4/CD8 ratio (p<0.001), 35% more CD8 activation (p = 0.02), 36% greater α4β7+ CD4 T cells (p<0.01), 54% more HLA-DR+ CD4 T cells (p<0.001), and 20% higher plasma VCAM (p<0.01) compared to CRs. The INR phenotype was not associated with levels of Kyn/Trp, CRP, TNF, IFNγ, IL-8, IL-6, sCD14, D-Dimer, I-FABP, MCP-1, ICAM or CD8%HLA-DR+.

**Conclusions:**

Peripheral CD4 non-recovery during long-term treated HIV infection is characterized by elevated CD8 activation and CD4 gut homing. Gut-focused interventions may be warranted in the INR context, and CD8 activation may serve as a surrogate endpoint for clinical interventions.

## Introduction

Effective antiretroviral therapy (ART) has dramatically improved life expectancy for individuals living with HIV. Despite this, HIV infection remains linked to an increased risk of premature death and serious non-AIDS health events (SNAE) such as cardiovascular events, non-AIDS cancers, and renal or liver disease [1]. SNAE are observed at a greater frequency among people living with HIV compared to the general population, with some suggestion that they occur at an earlier age [2].

The fundamental drivers of SNAE are poorly defined, but thought to be linked to chronic immune activation [3], which in turn may be a consequence of viral coinfections, low-level HIV replication, microbial translocation across the damaged mucosa of the gastrointestinal tract [4–6], antiretroviral therapy and/or lifestyle-related factors, such as smoking and high cholesterol [3, 7]. More recently, it has become clear that SNAEs disproportionately affect individuals with suboptimal immune restoration despite viral RNA suppression with ART. Specifically, a low number of blood CD4+ T cells (CD4 count) at the time of ART initiation, and slow recovery of blood CD4 count thereafter, have been associated with SNAE and premature death [2, 8–10].

The term immunologic non-responder (INR) has been used to describe individuals with incomplete recovery of CD4 counts in blood despite HIV RNA suppression on ART [9, 11]. There is no consensus on the clinical INR definition, with studies using a CD4 count upper peak between 200/mm^3^ and 500/mm^3^ despite prolonged virus suppression [9, 11]. The duration of viral suppression required to qualify for an INR definition also varies, with some studies defining individuals as INR if their blood CD4 count has not recovered as little as a year after treatment initiation [9]. It may be important to distinguish these early INR individuals from those with poor immune recovery despite many years of ART.

While the pathogenesis of the INR phenotype remains insufficiently characterized, it may involve a combination of peripheral inflammation, gut epithelial and immune damage, and abnormal immune cell gut homing [11–13]. Characterizing these mechanisms is essential to guide the development and evaluation of relevant therapies. Furthermore, inflammatory biomarkers that are specific to the INR phenotype may constitute surrogate endpoints for use in clinical intervention studies. The percent of surface co-expression of the activation antigens HLA-DR and CD38 on CD8+ T cells (CD8 activation) has been identified as a predictor of disease progression during untreated HIV infection [14]. CD38 expression on CD8+ T cells has also been described as a standalone marker of clinical disease progression in the untreated context [15]. Building on preliminary work from our group [16], we hypothesized specifically that increased CD8 activation would characterise the INR phenotype in the context of long-term suppressive ART, validating the marker for use in clinical trials. Further, we sought to characterize the immunology of the INR phenotype more broadly by assessing cellular and soluble measures of immune activation and gut immunopathology.

## Methods

### Study design and participant recruitment

This blinded cross-sectional cohort study included 101 participants from two HIV care sites in downtown Toronto, Canada: The Immunodeficiency Clinic at Toronto General Hospital (TGH) and The Maple Leaf Medical Clinic (MLMC). All participants were active patients recruited by clinic staff. Individuals with HIV were selectively recruited to establish a study cohort with a broad range of CD4 counts and CD4/CD8 ratios. To be included, participants with HIV had to have been on ART with HIV RNA suppression for at least two years. Exclusion criteria included active hepatitis B or C co-infection, pregnancy, use of chemotherapy, chronic steroid therapy or other immunomodulatory medications. Of 85 participants with HIV, one individual was excluded post-enrolment due to pregnancy, and a second due to sample processing failure. The reference group of HIV-negative participants were male clinic patients selectively recruited to ensure the group was of similar age to the group of people living with HIV. The study was approved by the University Health Network Ethics Review Board and all participants gave written informed consent prior to sample collection. Patient charts were manually reviewed, extracting data regarding demographics, antiretroviral agents, immune cell numbers, HIV RNA, intercurrent illnesses, and comorbidities.

### Clinical definitions

Individuals on suppressive antiretroviral therapy (ART) for at least two years with a peripheral blood CD4+ T cell count persistently <350/mm^3^ were defined as Immunologic Non-Responders (INR); Clinical Responders (CR) were defined as ART-treated individuals with a peripheral blood CD4+ T cell count >350/mm^3^.

### Laboratory processing and assays

Blood specimens were processed, cryopreserving plasma at -80°C. Peripheral Blood Mononuclear Cells (PBMCs) were processed into RPMI-1640 media containing 8.9% Fetal Bovine Serum (FBS), 89.29 u/mL penicillin, 89.29 ug/mL streptomycin, 1.79 mM L-glutamine (R10; Gibco), and stored at -150°C in FBS (Wisent Bioproducts) containing 10% DMSO (Fisher BioReagents). PBMCs were flash thawed in batches and incubated in R-10 at 37°C for 2 hours. Cells were then labelled in two antibody panels containing: BUV395-anti-CD45 (BD Horizon), ECD-anti-CD4 (Beckman Coulter), BV605-anti-CD3 (Biolegend), APC-H7-anti-CD8 (BD Pharmingen), AF700-anti-CD38 (BD Pharmingen), BV785-anti-HLA-DR (Biolegend), APC-anti-β7 (BD Pharmingen), Pac Blue-anti-CD3 (BD Pharmingen), PE-anti-α4 (BD Pharmingen), as well as LIVE/DEAD Aqua Dead Cell Stain (Invitrogen; L34957). Samples were analyzed with a LSRFortessa X-20 flow cytometer (BD Biosciences), ensuring inter-experiment consistency with calibrator beads (Spherotech URFP-30-2). Data were further analyzed using FlowJo (Treestar, Woodburn, Oregon, USA).

Commercially available ELISA kits were used, according to manufacturer guidelines, to quantify Intercellular Adhesion Molecule 1 (ICAM) (Invitrogen), Vascular Cell Adhesion Molecule 1 (VCAM), C-Reactive Protein (CRP), sCD14 (R&D Systems), D-Dimer (Sekisui Diagnostics), Intestinal Fatty Acid Binding Protein (I-FABP) (Hycult Biotech), with data collected using a SpectraMax190 Microplate Reader (Molecular Devices). Interleukin (IL)-6, IL-8, Interferon (IFN)-γ, Tumor Necrosis Factor (TNF), and Monocyte Chemoattract Protein 1 (MCP-1) were quantified using a multiplex immune assay (Meso Scale Discovery, Gaithersburg, Maryland, USA), with data collected using a QuickPlex SQ 120 Plate Reader (Meso Scale Discovery). Plasma tryptophan and kynurenine were quantified by LC-MS/MS as previously described.[17]

### Statistical analysis

Demographic and clinical characteristics of INRs and CRs were compared using Wilcoxon rank-sum tests for continuous variables and Chi-square or Fisher’s exact tests, as appropriate, for categorical variables. Separate multivariable linear regression models were used to estimate the association between INR phenotype and each plasma and cellular marker. To meet the assumptions for linear regression, biomarkers were log_10_ transformed and two outlier data points (one in dataset for each IL-6 and Kyn/Trp; as identified by residual diagnostics) were excluded from the analyses. Removal of outliers did not change the significance of statistical tests. Age, sex, duration of ART, and recent infection/vaccination (within 2 weeks) were chosen *a priori* to be included in the multivariable model. Model estimates were exponentiated to show the adjusted Ratio of Means (aROM) for the INR vs. CR phenotype.

## Results

### Participant characteristics

Participants with HIV (N = 83) were classified as either INR (N = 36) or Clinical Restorers (CRs; N = 47). HIV-negative participants (N = 18) were included for comparison. INR participants had a median CD4 count of 234/μl, compared to 618/μl in CRs (Table 1). INR individuals had taken ART for a median of 8 years, compared to 13 years for CRs (p = 0.179), and had a lower CD4/CD8 ratio (0.40 vs. 1.10 in CRs, p<0.001; Table 1). There was no difference between INRs and CRs in the components of their ART regimen (Table 1). INR individuals reported a higher prevalence of prior coronary syndromes, including unstable angina and myocardial infarction, but not of other comorbidities (Table 1).

**Table 1:** Participant characteristics.

	HIV-Positive Overall (IQR)	Clinical Responders (CD4 cell count >350/μl) (IQR)	Immune Non-Responders (CD4 cell count <350/μl) (IQR)	HIV-Negative (IQR)	p*
**N**	83	47	36	18	-
**CD4/mm^3^ blood**	436 (282,698)	618 (539,898)	234 (197,293)	655 (535,839)	<0.001
**CD4/CD8**	0.70 (0.40,1.20)	1.10 (0.70,1.48)	0.40 (0.29,0.50)	-	<0.001
**Age**	54 (8,59)	53 (48,59)	54 (50,58)	53 (47,58)	0.676
**Female [n (%)]**	17 (20.5)	12 (25.5)	5 (13.9)	0 (0)	0.274
**MSM [n (%)]**	57 (69.5)	33 (70.2)	24 (68.6)	18 (100)	0.999
**IDU [n (%)]**	3 (3.7)	2 (4.3)	1 (2.9)	-	0.999
**Current Smoker [n (%)]**	18 (21.7)	10 (21.3)	8 (22.2)	2 (11.1)	0.999
**Active or Resolved History [n (%)]**					
Hypertension	21 (26.6)	10 (22.2)	11 (32.4)	3 (16.7)	0.441
Hyperlipidemia	34 (42.0)	18 (39.1)	16 (45.7)	7 (38.9)	0.651
Acute Coronary Events	7 (8.8)	1 (2.2)	6 (17.6)	0	0.038
Hepatitis B (Resolved)	6 (7.6)	3 (6.7)	3 (8.8)	1 (5.6)	0.999
Hepatitis C (Resolved)	2 (2.5)	1 (2.2)	1 (2.9)	0	0.999
**Duration of HIV (y)**	16 (9,26)	16 (10,26)	15 (6,24)	-	0.355
**Duration on Combination ART (y)**	11 (5,18)	13 (7,18)	8 (3,18)	-	0.179
**ART Regimen Includes [n (%)]**					
II	50 (60.2)	29 (61.7)	21 (58.3)	-	0.823
NNRTI	32 (38.6)	14 (29.8)	18 (50.0)	-	0.072
PI	20 (24.1)	9 (19.1)	11 (30.6)	-	0.302

MSM, male who reported having had sex with men; IDU, reported injection drug use; ART, antiretroviral therapy; II, integrase inhibitor; NNRTI, non-nucleoside reverse transcriptase inhibitor; PI, protease inhibitor. HIV negative values included for reference only. *P values report confidence of comparison between CR and INR groups only; assessed, as appropriate, by Wilcoxon signed-rank, Chi square, or Fisher’s exact test.

### The INR phenotype is characterized by elevated T cell activation and gut homing

**Fig 1:**
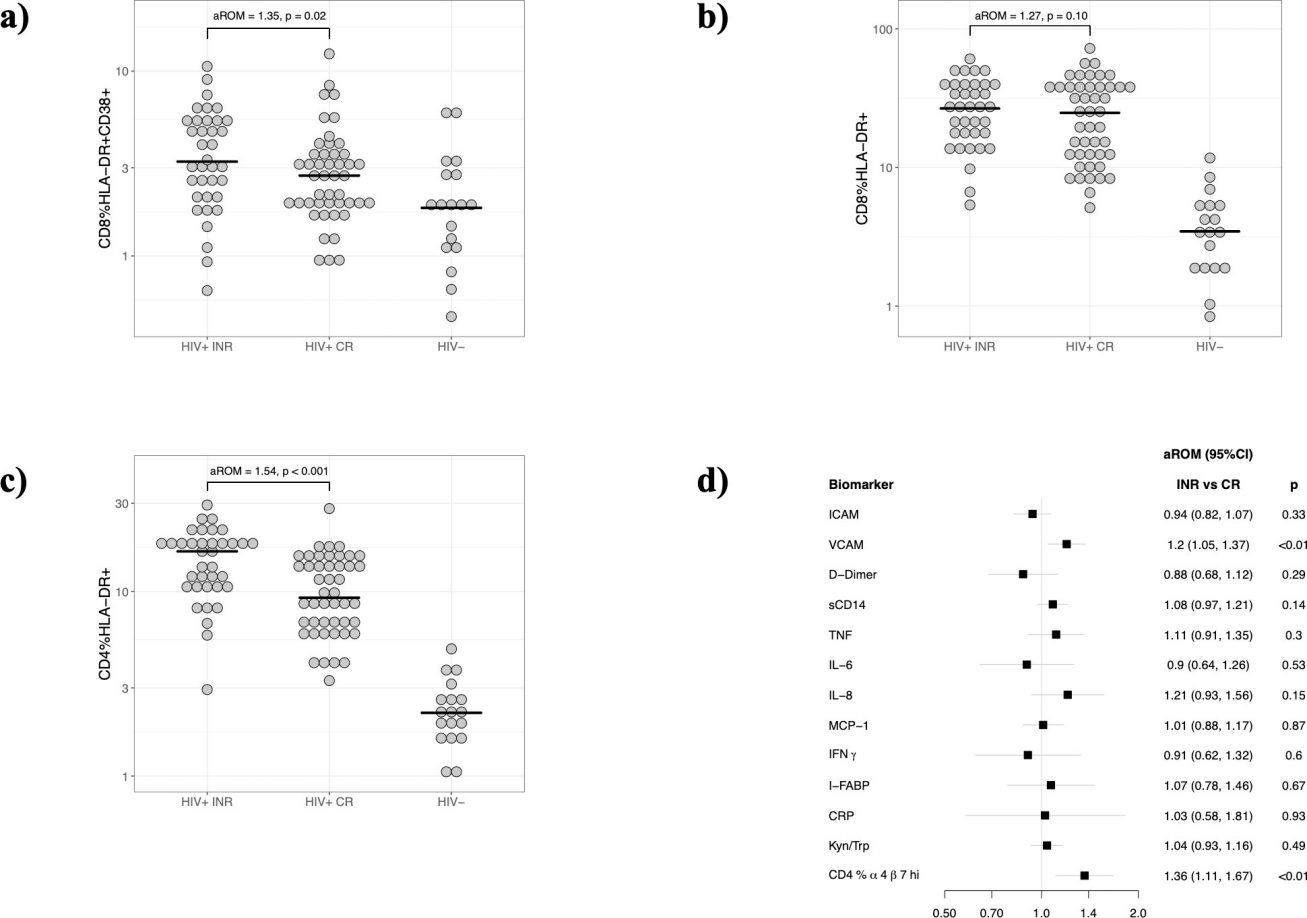
Characterizing immune differences between INR and CR groups. T cell activation was compared between INR, CR and HIV-uninfected groups based on (a) CD8+ T cell co-expression of HLA-DR and CD38; (b) CD8+ T cell expression of HLA-DR; and (c) CD4+ T cell expression of HLA-DR. Y-axis demonstrates % expression; the adjusted ratio of means (aROM) and p-value reflect a multivariable analysis controlling for age, sex, duration of ART, and recent infection/vaccination. (d) Additional analyses compared plasma and cellular markers of immune activation, gut damage, coagulopathy, and gut homing.

CD8 activation was defined as the proportion of CD38 and HLA-DR co-expression on CD8+ T cells. In models that were adjusted for age, sex, duration of ART, and recent infection/vaccination, the level of CD8 activation in INRs was significantly higher than CRs (aROM = 1.35; p = 0.02), although both HIV-infected groups demonstrated much higher levels of T cell activation than HIV-uninfected controls (Fig 1A). Interestingly, the proportion of CD8+ T cells expressing HLA-DR was not increased in the INR group compared to the CR group (p = 0.10; Fig 1B), while the proportion of CD4+ T cells expressing HLA-DR was 54% higher in the INR group (p<0.001; F 1c).

In addition, the proportion of CD4+ T cells expressing the integrin α4β7 (p<0.01), an integrin that mediates the homing of T cells to the gut mucosa, was increased in the INR group compared to the CR group (p<0.01; Fig 1D), and the proportion of CD8+ T cells expressing CD38 –but not HLA-DR–trended towards an increase of 25% in the INR group compared to the CR group (P = 0.07); Fig 1D).

### Elevated levels of plasma VCAM, but not other soluble immune factors, in INR participants

Having assessed T cell parameters, we next assayed plasma levels of several additional inflammation-related soluble biomarkers that had been previously linked to either treated or untreated HIV infection (see Methods, above). Soluble VCAM was the only plasma marker that was elevated in the INR group compared to CR individuals (Fig 1D). All other plasma biomarkers, including TNF, sCD14, CRP, ICAM, MCP-1, IFNγ, IFABP, D-Dimer, IL-8, IL-6, and Kyn/Trp showed no significant difference between INR and CR groups (Fig 1D).

## Discussion

Suboptimal recovery of CD4+ T cell numbers during HIV treatment has been associated with immune activation, premature death, and SNAE [8–10]. It remains unclear whether there is an ideal biomarker of inflammation which may help predict clinical outcomes in this population or which may serve in evaluating interventions. Our results demonstrate that CD8 activation, previously defined as a predictor of disease progression [14], is significantly elevated in INR individuals compared to clinical restorers. Interestingly HLA-DR expression, another marker of immune activation, was substantially increased on CD4+ but not CD8+ T cells in the INR group. This suggests that, while CD8 activation does characterize the INR phenotype, CD4+ T cell immune alteration may also play a central role in INR-associated immune dysfunction. Importantly, other commonly measured biomarkers of SNAE risk were elevated but not INR-specific, emphasizing the need to validate the relevance of inflammatory biomarkers for use in distinct clinical populations. More, INR individuals in our cohort were more likely to have experienced coronary events, supporting the notion that CD8 activation may predict clinical outcomes.

Multiple mechanisms are hypothesized to contribute to the persistent inflammation during HIV in the context of suppressed viremia, including: (1) low-level virus replication in tissues; (2) intermittent reactivation of co-pathogens, such as herpesviruses; (3) damage to gut epithelial integrity by HIV, allowing bacterial products to transit into the bloodstream [18]; and (4) dysfunctional immunopathogenic feedback loops, implicating immunoregulatory pathways and contributing to tissue fibrosis.[3] In agreement with other studies of INR populations [11, 19], our findings, including elevated peripheral CD4+ T cell activation and integrin α4β7 expression, suggest that there may be a relationship between cellular immune activation and homing of lymphocytes to the gut. Interestingly, others have found that CD4+ T cells expressing CCR9 and α4β7 may remain in the blood of ART-treated people rather than homing to the gut, perhaps due to reduced gut mucosal expression of the CCR9 ligand CCL25 [20]. This suggests that exploring gut-targeted research and/or interventions to enhance T cell gut homing and mucosal immune reconstitution may provide an opportunity to improve health outcomes in this population. Furthermore, assessment is warranted of a link between CD8 activation, CD4+ T cell expression of HLA-DR, and upstream or by-product immunopathogenesis, such as through T cell exhaustion or metabolic dysfunction.

Our study has potential shortcomings that merit discussion. Our sample size was modest, and so the significant relationship demonstrated between the INR phenotype and a set of immune-related measures does not rule out the potential biological meaningfulness of uncaptured less-dramatically-elevated markers. Although we demonstrated an increased signal of lymphocyte homing to the gut, we found no concurrent increase in plasma sCD14, a microbial translocation and gut damage associated marker [18], leaving this signal unexplained and worthy of further exploration. Specifically, it remains unclear whether increased gut-homing of peripheral T cells somehow confounds the INR phenotype, or whether these biomarkers occur downstream of an INR phenotype development. Further, our study did not include a sensitivity analysis to explore potential relationships amongst ART regimen components, ART adherence, and measured biomarkers. Our population was from a single urban centre and largely comprised men who have sex with men on ART for many years, and so generalizability to other populations will need to be demonstrated. While there were significant differences in inflammatory biomarkers between the CR and INR groups, there was substantial heterogeneity with no clear cut off; while this means that these inflammatory biomarkers are unlikely to constitute a future diagnostic tool, the peripheral blood CD4+ T cell count is sufficient for this purpose, while our goal was to investigate clues regarding pathogenesis and potential clinical impact. Finally, non-significant differences between INR and CR groups in some biomarkers, including sCD14 and TNF, suggest that larger and therefore better powered future studies may reveal additional biomarkers associated with reduced immune reconstitution.

Overall, this study achieved its goal of validating CD8+ T cell activation, previously linked to disease progression in ART-naïve individuals [14], as a surrogate marker of the persistent immune activation that characterizes HIV-associated suboptimal immune restoration. Elevated CD4+ T cell expression of HLA-DR may also serve as a biomarker for the INR phenotype, and the underlying pathogenesis of this elevation merits further study. Further research is warranted to assess whether clinical interventions–especially those focused on improving gut immune health–can decrease INR-associated CD8+ T cell activation, and ultimately whether such interventions improve clinical outcomes.

## Supporting information

S1 File (XLSX)
